# 

**Published:** 1869-01

**Authors:** 


### INDEX TO VOLUME XXIII.

A Plea for Specialties 14
An Experience 18
Address at the commencement of the Ohio College of Dental Surgery 53
A New Alloy of Alluminum 88
Artificial Dentures 97
A Wide Circuit 130
American Dental Association 138
Assaying Silver Compounds in the wet way 178
Atlas to the Pathology of the Teeth 183
Address 185
A Case of Petrifaction 225
A Superior Liquid Glue 225
Alveolar Abscess 253
Anæsthetics Adverse to Union by First Intention 253
Assimilation of Phosphate of Lime and its Therapeutical Employment 254
Alcohol as a Dressing to Wounds 255
A New Growing Slide 256
A Speech 265
Another Rubber Patent 268
A Puff 316
Address of Dr. W. W. Allport 341
An Improved Syringe Pipe for Hypodermic Injection 355
Amaurosis Caused by Crowding of Teeth 353
Articulation 365
Alluminum 468
Advertisements of Specialists 474
A New Silver Alloy 474
A Model Document 476
An Improved Compound for Welding 477
A Definition 478
A Worthy Foundation 481
A Collection of Pins 481

Annual Address before the Ohio State Dental Society 493
Amalgam, and its Proper Use 502
A Peculiar Case 522
American Academy of Dental Science 524
A Key to every Imposture and Villainy 532
Brown—Sequard 134
Bromide of Patassium for the Troubles of Teething 351
Bromide of Potassium Pushed to its Full Extent 483
Bibliographical 488
Correspondence 37, 64, 162, 309, 485
Continuous Electrical Currents in the Treatment of the Suspension of Vital Actions Caused by Chloroform 43
Commencement 51, 182
Chloro-Nitrous Oxyd 60, 194
Calcification of Tooth Pulp 92
Carbolic Acid Treatment in Surgery 130
Coffee as a Deodorizer 134
Continuous Gum Work 135
Constitution of the American Dental Association 214
Contour Fillings 237
Chloroform inhalations in Strychnia Poisoning 253
Classification of Boston Doctors 256
Calcification of Tooth Pulp 264
Contour Fillings—When are they Indicated? 276
Capping Exposed Pulps 280
Carbolic Acid, and its Therapeutical Uses 299
Chart of the Physiological Arrangement of Cranial Nerves 311
Correction 349
Cleaning the Teeth 361
Cleft Palate 389
Chloral 438
Chromic Acid 442
Chloro Nitrous Oxyd 516
Close of the Volume 536
Dentists in Belgium 40
Dental Depot—A Change 50
Depression and Replacement of the Superior Maxilla 89
Do we Use Os-Artificial? 140
Difference of Excretion of Carbonic Acid and Absorption of Oxygen under Different Circumstances 165
Diseases of the Jaws 168
Detection of Mercury in Cases of Poisoning 176
Dental Squibs 188
Dental Association 223
Dental Hygiene 229

Death from Hypodermic Injection 263
Difficulties in Practice 315
De-Nicotinized Tobacco 385
Dental Ethics 405
Disarticulation of the Upper Maxillary Bones 432
Detection of Blood Stains 438
Dental Luminary 490
Editorial 49, 91, 135, 181, 227, 264, 312, 359, 399, 443, 488, 536
Ethics 317
Educational 489, 447
East Tennessee Dental Association 467
Floral Guide 52
Flexible Sulphur 84
Forest City Society of Dental Surgeons 250
Fatal Case of Tetanus 350
Food in the Reign of Henry the Seventh 358
Facial Neuralgia 389
Found 403
Fracture of the Superior Maxillary Bone 479
Hyperdermic Medication 81
How to Count the Lines in Robert's Plates 179
Harris Dental Society 213
Health of Dentists 235
Hypophosphites in the Toothache of Pregnancy 255
Hyperdermic Method of Injection 259
How to Conduct a Dental Practice 331
Hudson River Association of Dental Surgeons 348
Hare Lip 390
Hydrogenium 392
Hypertrophy of Nails and Bones 442
Hygiene in its Relation to Therapeutics 526
Influence of Diet upon the Mother's Milk 40
Imperfect Enamel 62
Influence of Medicaments on the Heart 178
Iodine and Aconite in Periodontitis 254
Insanity Cured by the Removal of Carious Teeth 353
Influence of Tobacco upon the Circulatory System 356
In Toe-Toe 400
Introductory Address to the 24th Annual Session of the Ohio College of Dental Surgery 449
Influence of Education 469
Japanese Dentistry 262
Keeping Volatile Liquids 388
Keenness of the Senses 482
Knoxville 485

Leeches 226
Library of the American Medical Association 478
Library of the Ohio College of Dental Surgery 489
Mechanical Dentistry—Past and Present 9
Meeting of the Chicago Microscopical Association 94
Meeting of Dentists—Resolutions of Respect 96
Manipulating Foil 190
Microscopy of the Dental Tubuli 324
Miscellanies 413
Microscopy of a Tooth 498
New York State Dental Society 75
New Modes of Preparing Objects for the Microscope 84
Ney's Battery 134
Notes from L'Union Medicale 174
North Iowa Dental Association 180
Northern Ohio Dental Association 245
New Uses for Mica 254
New Mode of Preparing Objects for the Microscope 258
Nitrous Oxyd 312
New Operation in Dental Science 440
Odontomes 44
Ohio State Dental Association 64
Obituary 96, 184, 536
Original Communications 141, 185, 229, 273, 317, 361, 405, 449, 97
Operative Dentistry 243
On the Use of Chloride of Gold in Microscopy 257
Official Proceedings of the Dental Convention, Atlanta 368
On the Structure of Two Forms of Tooth Tumor 372
On the Therapeutic Action of Aconite and its Preparations 382
On the Renouncing of Rubber by the Dental Profession 422
On the Medicinal Use of Phosphorous and its Compounds 434
Organic Synthesis 442
Odds and Ends 459
Os-Artificial Impugned 461
Ohio State Dental Society 491
Os-Artificial Impugned 519
Preservation of Animal Tissues 41
Proceedings of Societies 67, 121, 197, 245, 288, 345, 368, 524
Purification of Tannin 358
Peculiar Dental Practice 367
Personal 404
Prof. Boehm—Dissection Wound 478
Phosphoric Acid in Organic Nature 482
Progress 490
Report of Discussions at the Ohio State Dental Society 22

Record 45
Rose Pearl 49
Root Filling 161
Rimming Plates 194
Report of Discussions at the 24th Annual Meeting of the Miss. Valley Dental Association 288, 345, 197
Resignation and Appointment 359
Rich Beef Tea 535
Sub-Periosteal Resection 46
Selections 40, 77, 127, 165, 224, 252, 299, 350, 372, 430, 469, 526
Suppuration 58
Southern Dental Association 64
Sore Gums or Throat 88
Some Remarks on Metal Plates 160
Sulphate of Nickel—A Sedative 224
Sore or Sour? 227
Suspension of the New York College of Dentistry 270
Supreme Judicial Court 271
Sponge Tents 308
Spectrum Lines 357
Sensitive Dentine in a Pulpless Tooth 360
Southern Dental Association 404
Scarifying the Gums 483
Specialties 530
The Microscope 41
Test for Pure Glycerin 47
Testing Water for Organic Impurities 47
The Fee for Testifying as Expert 48
The Miss. Valley Dental Association 51
The Ohio Dental College Association 52
The New Webster 80
The Principles Regulating the Natural Evacuation of Abscesses 70
The Therapeutics of Wakefulness 85
The Present Status of the Rubber Question 91
The Rubber Dam 93
The Health of the Dentist—Means of Preserving 108, 147, 181
The Twenty-fifth Annual Meeting of the Miss. Valley Dental Asso. 121
The Treatment of Neuralgia by Electrization 127
The Action of Belladonna 131
To Ascertain the Power of a Microscope 133
The First Inoculation for Small Pox 133
The First Quack in America 133
The First Persons Vaccinated in America 134
The Physician's Dose and Symptom Book 140
The Dental Profession, *vs.* Rubber 141

Tinting Vegetable Tissues ... 176
Temperature of the Body in Health ... 177
The First Physician in Massachusetts ... 179, 258
The Mad River Dental Society ... 184
To Prevent Vulcanizers from leaking steam ... 192
Two Cases of Death from Chloroform ... 225
Third Dentition ... 224
Treatment of Diseased Gums ... 226, 259
The Last Wonder of the Spectroscope ... 226
The Hands ... 242, 364
The Administration of Chloroform ... 252
The Boston Dental College ... 271
Thoughts and Experiences on Chloro-Nitrous Oxide for One Week ... 273
The Eleventh Annual Meeting of the Indiana State Dental Asso. ... 293
The Nerves of the Heart ... 357
Tooth Powders, Etc ... 359
Taking Impressions for Partial Sets of Teeth ... 386
The Highest Branch of Medicine ... 437
The Food of a Human Being ... 475
The Author of Thesaurus ... 473
Tooth Brushes ... 475
The Earliest Writer on Carbolic Acid ... 476
The Practice of Medicine in Oregon ... 484
To Whom it May Concern ... 487
The New Masterpiece ... 491
To the Members of the American Dental Association ... 492
Treatment of Tooth Pulps ... 511
Use of Chloride of Gold ... 436
Use of Os-Artificial ... 443
Valedictory Address to the Graduating Class, O. D. C. ... 104
Vice Versa ... 489
Vegetable Nature of Diatomacæ ... 552
Was the Nerve regenerated ? ... 48
When should the File be Used ? ... 157
Western New York Dental Association ... 179
Wabash Valley Dental Society ... 212
What are the Indications for Extraction ? ... 284
Worse than the Wicked Flea. ... 445
Weights and Measures ... 477

### OHIO COLLEGE

OF

### DENTAL SURGERY.

The Twenty-third Annual Session of the Ohio College of Dental Surgery will commence on Tuesday the 15th of October, and continue five months.

### FACULTY.

JAMES TAYLOR, M. D., D. D. S.,
Emeritus Professor of Institutes of Dental Science.

C. KEARNS, M. D.,
Professor of Anatomy.

GEO. E. JONES, M. D.,
Professor of Pathology.

GEORGE WATT, M. D., D. D. S.,
Professor of Chemistry and Therapeutics.

EDWARD RIVES, M. D.,
Professor of Physiology and Histology.

J. TAFT, D. D. S.,
Professor of Operative Dentistry and Dental Hygiene

H. R. SMITH, M. D., D. D. S.,
Professor of Mechanical Dentistry and Metallurgy.

J. A. WATLING, D. D. S.,

Professor of Clinical Dentistry.

WILL TAFT, D. D. S.
Demonstrator of Anatomy.

### TEXT BOOKS.

Gray's and Wilson's Anatomy; Dalton's or Carpenter's Physiology; Williams' Pathology; Fown's, Turner's, Graham's, or Brandt and Taylor's Chemistry; Taft's Operative Dentistry; Richardson's Mechanical Dentistry; Harris' Principles and Practice of Dental Science, Tomes Dental Surgery, Watts' Chemical Essays, Harris' Dental Dictionary.

### TERMS OF ADMISSION.

Tickets must be procured at the beginning of the session.

| | |
|---|---|
| Tickets for the course, | $100 00 |
| Demonstrator's Ticket for Anatomy, | 5 00 |
| Matriculation Fee, but once, | 5 00 |
| Diploma Fee, | 30 00 |

### TERMS OF GRADUATION.

Two courses, (including one of dissections,) with a satisfactory dental pupilage.

Eight years reputable practice, or a satisfactory examination upon the branches of the Junior Course in this Institution will be received as equal to one course.

An acceptable thesis upon some subject on Dental science.

Must possess a good moral character, and be twenty-one years old.

For further information, address J. TAFT, *Dean.*
117 West Fourth Street

AMERICAN DENTAL ASSOCIATION—Meets annually (next year at Saratoga. *Pres.* J. TAFT, of Cincinnati, O. *Rec. Sec.* EDGAR PARK, of St. Louis, Mo.

*List of Societies represented in the American Dental Association.*

ALBANY DENTAL ASSOCIATION—Meets monthly at Albany. *Pres.* ROBERT NELSON, *Rec Sec.* W. F. WINNE, *Cor. Sec.* B. WOOD, of Albany.

AMERICAN ACADEMY OF DENTAL SCIENCE—Meets at Boston, Mass, *Pres.* DANIEL HARWOOD, M. D., of Boston. *Sec.* E. N HARRIS, D, D. S., of Boston.

BUFFALO DENTAL SOCIETY—Meets monthly at Buffalo. *Pres.* G. C. DA BOLL, *Rec Sec.* S. A. FREEMAN.

BROOKLYN DENTAL ASSOCIATION—Meets semi-monthly *Pres.* W. C. PARKS, *Rec. Sec.* WM. C. HORNE, of Brooklyn.

CINCINNATI DENTAL SOCIETY—Meets semi-monthly at Cincinnati. *Pres.* Geo. WATT, *Rec. Sec.* Wm. TAFT.

CENTRAL OHIO DENTAL ASSOCIATION—Meets Semi-annually on the second Tuesday of May and November. *Pres.* Dr. M. DECAMP, Mansfield, O., *Sec.* M. A. SPENCER, Daylstown, O.

CENTRAL STATES DENTAL ASSOCIATION—Meets semi-annually. Next meeting at Indianapolis. The Annual meeting at Louisville, Ky., during the Holidays. The Semi-annual on the first Tuesday of April. *Pres.* Dr. J. A. McCLELLAND, *Sec.* J. F. CANINE, Louisville, Ky.

CENTRAL NEW YORK DENTAL ASSOCIATION. Meets semi-annually. *Pres.* S. B. PALMER, *Rec. Sec.* S. G. MARTIN, *Cor. Sec.* P. HARRIS of Skaneatles.

CHICAGO DENTAL SOCIETY—Meets monthly at Chicago. *Pres.* M. S. DEAN, *Rec. and Cor. Sec.* A. W. FREEMAN, of Chicago.

CONNECTICUT VALLEY DENTAL ASSOCIATION—Meets semi-annually, second Tuesday of May and October, *Pres.* A. A. HOWLAND, Barre, Vt. *Rec. Sec.* W, H. JONES, of Boston, Mass.

CONNECTICUT STATE DENTAL ASSOCIATION—*Pres.* W. W. SHEFFIELD, New London. *Cor. Sec.* JOHN CODY, Hartford. *Rec. Sec.* WM. ALLENDER, New London.

DELAWARE DENTAL ASSOCIATION—Meets semi-annually. *Pres.* Dr. SMITH. *Cor. Sec.* SAMUEL MARSHALL,

EAST TENNESSEE DENTAL ASSOCIATION—Meets annually at Knoxville, Tenn *Pres.* J. FOUCHE. of Knoxville *Rec. Sec.* W. H. COOK, of Cleveland, Tenn.

† HARRIS DENTAL ASSOCIATION OF LANCASTER, Pa.—*President*, JOHN McCALLA. *Sec.* WM. NICHOLS AMER.

HUDSON RIVER ASSOCIATION OF DENTAL SURGEONS—Meets on the third Thursdays of May, September and January. *Pres.* E. D. FULLER, Peekskill. *Rec. Sec.* L. S. STRAW, Newburg.

HUDSON VALLEY DENTAL ASSOCIATION—meets monthly at Troy, N. Y. *Pres.* S. P. WELCH, Lansingburgh, *Rec. Sec.* E. J. YOUNG, *Cor. Sec.* L. C. WHEELER, of Troy.

ILLINOIS STATE DENTAL SOCIETY—Meets annually. *Pres.* E. H. KILBOURNE, of Aurora, Ill. *Sec* H. J. SMITH, Princeton, Ills.

INDIANA STATE DENTAL SOCIETY—Meets annually at Indianapolis on the las Tuesday of June. *Pres.* S. M. GOODE, of Madison. *Rec. and Cor. Sec.* E. M. MORRISON of Noblesville.

IOWA STATE DENTAL SOCIETY—Meets semi-annually Jan. and July. Next meeting at Muscatine. *Pres.* L. C. INGERSOLL, of Keokuk. *Rec. Sec.* Wm. O. KULP, of Muscatine. *Cor. Sec.* N. H TULLOSS, of Iowa City.

LAKE ERIE DENTAL SOCIETY.—Meets on the 4th February at Girard, Pa. *Pres.* A. B. ROBBINS.

LEBANON VALLEY DENTAL ASSOCIATION—*Prest.* W. K. BRENIZER, *Sec.* S. H. GUILFORD.

LOUISVILLE DENTAL SOCIETY—Meets on the first Tuesday of each month. *Pres.* ———, *Cor. Sec.* W. H. SHADOAN.

MAD RIVER VALLEY DENTAL SOCIETY—Meets quarterly. Next meeting in Dayton, on the first Tuesday of October next. *Pres.*, B. F. ROSSON, *Sec.*, G. L. PAINE.

MARYLAND ASSOCIATION OF DENTISTS—Meets the last Thursday of every month. *Pres.*, J. B. BEAN, *Sec.*, S. M. FIELD .

MASSACHUSETTS DENTAL SOCIETY—Meets monthly. *Pres.*, E. G. LEACH, *Sec.*, D. G. HARRINGTON, Boston.

MICHIGAN DENTAL SOCIETY—Meets annually second Tuesday of Oct. at Detroit. *Pres.* J. A, Harris, of Pontiac *Rec. and Cor. Sec.* GEO. LEARY, Adrian.

MISSISSIPPI VALLEY DENTAL ASSOCIATION -Meets [illegible] at Cincinnati, *Pres.* GEO H. CUSHING, of Chicago, Ill. *Rec. Sec.* WILL TAFT, *Cor. Sec.* M. WELLS, of Indianapolis, Ind.

MISSOURI DENTAL ASSOCIATION—Meets on the first Tuesday of June, in St. Louis. *Pres.* Dr. H. J. MCKELLOPS. St. Louis, *Sec* H. JUDD, St. Louis.

MERRIMAC VALLEY DENTAL SOCIETY—Meets semi annually first Tuesday of Oct, and May. *Pres.* A. LAWRENCE, *Rec. Sec.* G. A. GERRY, of Lowell.

MEMPHIS DENTAL SOCIETY.—Meets on the first Tuesday of each month. *Pres.* W. T. Arrington, *Sec.* V. F. Elliott. *Treas.* C. L. Harris.

NASHVILLE DENTAL ASSOCIATION—Meets on the second Tuesday of each month. *Pres.* W. H. MORGAN, *Sec* J. C. ROSS, *Cor. Sec.* R. RUSSELL.

NEW YORK SOCIETY OF DENTAL SURGEONS—Meets semi-monthly in New York. *Pres.* J. C. ROBBINS, *Rec. Sec.* J. S. LATIMER, of New York. *Cor. Sec.* W. N. ALLEN, 18 W. 11th street, New York.

NEW YORK STATE DENTAL SOCIETY—Meets at Albany on the last Tuesday of July next. *Pres.* A. WESTCOTT, of Syracuse. *Sec.* L. W. ROGERS, of Utica.

NEW HAVEN DENTAL SOCIETY—Meets on the first Wednesday of each month at New Haven. *Pres.* J. T. METCALF, *Rec. and Cor. Sec.* C. L. SMITH, of New Haven.

NEW LONDON COUNTY DENTAL SOCIETY—Meets monthly.* *Pres.* R. W. BROWNE. *Rec. Sec.* WM. ALLENDER

NORTHERN OHIO DENTAL ASSOCIATION—Meets Semi-annually, (first Tuesday of May and October.) in Cleveland. *Pres.* W. P. HORTON, *Rec. Sec.* H L. AMBLER, *Cor. Sec.* CHAS. BUFFETT, Cleveland.

NORTHERN IOWA DENTAL ASSOCIATION—Meets annually, next meeting second Tuesday of June. 1868, at Cedar Rapids. *Pres.* C. R. STERNEMAN, Cedar Rapids. *Rec. Sec.* P. C. BRANCH, Dubuque.

OHIO DENTAL COLLEGE ASSOCIATION—Meets annually March 13th, at Cincinnati. *Pres.* W. P. NORTON Cleveland, O. *Rec Sec.* J. TAFT, of Cincinnati.

OHIO STATE DENTAL ASSOCIATION.—Meets semi-annually, at Columbus. [Next meeting—first Tuesday of December.] *Pres.* W. P. HORTON, Cleveland, *Rec. Sec.* G. W. KEELY, Oxford, O. *Cor. Sec.* C. R. BUTLER, Cleveland, O.

ODONTOGRAPHIC SOCIETY OF PENNSYLVANIA—Meets monthly in Philadelphia *Pres.* J. H. MCQUILLEN, *Rec. Sec.* T. C. STELLWAGON, *Cor. Sec.* C. A. KINGSBURY, of Phila

ODONTOLOGICAL SOCIETY OF NEW YORK—Meets the second Tuesday of each month.* *Pres.* C. E. FRANCIS *Rec Sec.* THOS. BURGH.

OLD COLONY DENTAL ASSOCIATION—Meets quarterly.* *Pres.* D. S. DICKERMAN. *Rec. Sec.* GEORGE R. WHITNEY, *Cor. Sec.* L. W. PUFFER, North Bridgewater.

PENNSYLVANIA ASSOCIATION OF DENTAL SURGEONS—Meets monthly in Phila *Pres.* W. W. FOUCHE, *Rec. Sec* JAMES TRUMAN *Cor. Sec.* G. W. ELLIS, of Philadelphia

PITTSBURG DENTAL ASSOCIATION—Meets monthly, (second Tuesday, in Pittsburg *Pres.* M. E. GILLESPIE, *Rec. Sec.* J. D. WHITE.

PENINSULAR DENTAL SOCIETY—Meets quarterly.* *Pres.* SAMUEL MARSHALL, *Re Sec.* W. G. A. BONWILL, *Cor. Sec.* S. S. NONES.

POUGHKEEPSIE DENTAL SOCIETY.—Meets monthly, *Pres.* J. H. Mann, *Sec.* H. F Clark.

ST. LOUIS DENTAL SOCIETY—Meets monthly. *Pres.* W. H. EAMES. *Sec.* G. A. BOWMAN.

SUSQUEHANNA DENTAL ASSOCIATION—Meets semi-annually*. (Next meeting at Lewisburg. Pa., January 13, 1869.) *Pres.* C. S. BECK, M. D. *Rec. Sec.* R. C. BURLEM.

TENNESSEE DENTAL ASSOCIATION.—Meets semi-annually. *Pres.* W. T. Arrington, of Memphis, *Sec.* Wm M. R. Johns, of Somerville.

WASHINGTON CITY DENTAL ASSOCIATION—Meets annually. Dec. 2d. *Pres.* R. F. HUNT. *Sec.* H. B. NOBLE.

WESTERN DENTAL SOCIETY—Meets annually.* *Pres.* T. P. ABELL, *Rec. Sec.* C. W SPALDING, *Cor. Sec.* E. A BOGUE.

WESTERN NEW YORK DENTAL ASSOCIATION—Meets semi-annually.* *Pres.* L. J. WALTER, *Sec* W. C. BARRETT, Warsaw, N. Y.

AMERICAN DENTAL CONVENTION—Meets annually first Tuesday of March, (next meeting at New Haven,) *Pres.* J H. AMBLER, *Rec. Sec.* W. C. HORNE. *Cor Sec.* J. S. LATIMER, of New York.

†CUMBERLAND VALLEY DENTAL SOCIETY—Meets semi-annually.* *Pres.* J. L. SUESSEROTT, of Chambersburg *Sec.* GEO. W. NEIDICH, of Carlisle, Pa.

**Place of meeting fixed by appointment.*

† Not represented in the Am. Dental Association.

### LIST OF SOCIETIES REPRESENTED IN THE AMERICAN DENTAL ASSOCIATION.

BROOKLYN SOCIETY OF DENTAL SCIENCE AND ART*—*Pres.* H. G. MERICK. *Rec Sec.* E. L. CHILDS. *Cor. Sec.* THOS. JARVIE, Jr.

### DISTRICT DENTAL SOCIETIES OF THE STATE OF NEW YORK.†

*1ST DISTRICT DENTAL SOCIETY.—*Pres.* A. L. Northrop, New York City. *Sec.* W. C. Horne, New York City.

*2D DISTRICT DENTAL SOCIETY.—*Pres.* W. B. Hurd, Brooklyn. *Rec. Sec.* Wm. Jarvis Jr., Brooklyn. *Cor. Sec.* T. S. Straw, Newburg.

3D DISTRICT DENTAL SOCIETY.—Meets at Albany semi-annually and annually. Next meeting 2d Tuesday in July.—*Pres.* H. H. Young, Troy. *Sec.* W. F. Winne, of Albany.

*4TH DISTRICT DENTAL SOCIETY.—*Pres.* F. C. Rich, Saratogo Springs. *Sec.* C. A. Elmore, Fort Edwards.

*5TH DISTRICT DENTAL SOCIETY.—*Pres.* G. A. Foster, Utica *Sec.* Charles Barnes, Syracuse.

*6TH DISTRICT DENTAL SOCIETY.—*Pres.* H. S. McCall, Binghampton. *Sec.* T. A. Todd, Cortland.

*7TH DISTRICT DENTAL SOCIETY.—*Pres.* F. French, Rochester. *Sec.* John L. Clark, Waterloo.

*8TH DISTRICT DENTAL SOCIETY.—*Pres.* B. T. Whitney, Buffalo. *Rec. Sec.* W. C. Barrett, Warsaw. *Cor. Sec.* Geo. B. Snow, Buffalo.

* Place and time of meeting fixed by appointment.
† Not represented in the American Dental Association.

### EXCHANGES,

ST. LOUIS MEDICAL AND SURGICAL JOURNAL,
BOSTON MEDICAL AND SURGICAL JOURNAL,
THE PACIFIC MEDICAL AND SURGICAL JOURNAL, SAN FRANCISCO.
BUFFALO MEDICAL AND SURGICAL JOURNAL,
PHILADELPHIA MEDICAL AND SURGICAL REPORTER.
CINCINNATI LANCET AND OBSERVER,
DENTAL COSMOS, PHILADELPHIA.
L'ART DENTAIRE, PARIS, FRANCE.
BRAITHWAITE'S RETROSPECT, N. Y.
THE DENTAL TIMES, PHILADELPHIA,
LADIES' REPOSITORY, CINCINNATI.
HALL'S JOURNAL OF HEALTH, N. Y,
CHICAGO MEDICAL EXAMINER.
XENIA TORCHLIGHT.
THE DETROIT REVIEW OF MEDICINE AND PHARMACY.
MEDICAL RECORD, N Y.
NASHVILLE JOURNAL OF MEDICINE AND SURGERY,
AMERICAN ECLECTIC MEDICAL REVIEW, N. Y.
AMERICAN JOURNAL OF DENTAL SCIENCE, BALTIMORE.
THE UNITED STATES MEDICAL AND SURGICAL JOURNAL, CHICAGO,
NEW ORLEANS JOURNAL OF MEDICINE.
WESTERN JOURNAL OF MEDICINE. INDIANAPOLIS, IND.
AMERICAN AGRICULTURIST, N. Y.
DENTAL OFFICE AND LABORATORY, PHILA,
BOSTON JOURNAL OF CHEMISTRY.
CINCINNATI MEDICAL REPERTORY.
CHICAGO MEDICAL INVESTIGATOR.
JOURNAL OF APPLIED CHEMISTRY, N. Y.
DRUGGISTS' CIRCULAR AND CHEMICAL GAZETTE, NEW YORK,
DER ZAHNARZT, LEIPZIG.
DEUTSCHE VIERTELJAHRSSCHRIFT, NUREMBURG.
CANADA JOURNAL OF DENTAL SCIENCE, MONTREAL.
GUARDIAN OF HEALTH AND EDUCATION, BOSTON.
HARDWICKE'S SCIENCE GOSSIP, LONDON, ENGLAND.
PACKARD'S MONTHLY, NEW YORK.
POPULAR SCIENCE REVIEW, LONDON, ENGLAND.
MISSOURI DENTAL MONTHLY ST. LOUIS.
MEDICAL BULLETIN, BALTIMORE, MD.
ECLECTIC MEDICAL JOURNAL, CINCINNATI.

### THE MISSISSIPPI VALLEY DENTAL ASSOCIATION

Will hold its regular annual meeting, in the rooms of the Ohio Dental College, on Wednesday, the 3d day of March, beginning at ten o'clock A. M. We trust there will be a large gathering of the profession at that time. The meetings of this Society are always the occasion of much professional interest, and we hope the approaching meeting will be of equal, if not greater usefulness than those that have preceded it. The essays and discussions in this Society have, in a marked degree, embraced and blended the scientific and practical features of the profession. We shall hope to meet all of its old friends and all its new ones, and those who wish to become such.

### COMMENCEMENT.

The Commencement Exercises of the Ohio College of Dental Surgery will be held on the evening of March 3, in the lecture room of the College. An address will be delivered by the President of the Board of Trustees, and other interesting exercises appropriate to the occasion will take place. All who feel an interest in the Institution and its work are most cordially invited to be present.

### FLORAL GUIDE.

### THE OHIO DENTAL COLLEGE ASSOCIATION

Will hold its annual meeting, on Tuesday, March 2, at ten o'clock A. M., at which time it is very desirable that all the stockholders be present, as matters intimately connected with the interests of the profession, as well as this Institution, always come before this Association for its consideration. There is each year three members of the Board of Trustees to be elected, and other matters of importance to receive attention. We hope all the friends of the College may be present.

### CONTENTS.

ORIGINAL COMMUNICATIONS.

Mechanical Dentistry—Past and Present........ 9
A Plea for Specialties........ 14
An Experience........ 18

PROCEEDINGS OF SOCIETIES.

Report of Discussions at the Ohio State Dental Society, Dec., 1 and 2, 1868........ 22

Correspondence........ 37

SELECTIONS.

Influence of Diet upon the Mother's Milk........ 40

Dentists in Belgium........ 40

Preservation of Animal Tissues........ 41

The Microscope........ 41

Continuous Electrical Currents in the Treatment of the Suspension of Vital Actions Caused by Chloroform........ 43

Odontomes........ 44

Ricord........ 45

Subperiosteal Resection........ 46

Test for Pure Glycerine........ 47

Testing Water for Organic Impurities........ 47

Was the Nerve Regenerated........ 48

The Fee for Testifying as Expert........ 48

EDITORIAL.

Rose Pearl........ 49

Dental Depot—A Change........ 50

The Mississippi Valley Dental Association........ 51

Commencement........ 51

Floral Guide........ 52

The Ohio Dental College Association........ 52

---

### The Dental Register

OF THE WEST.

*Issued Monthly, at $3 a Year in Advance.*

DEVOTED TO THE INTERESTS OF THE DENTAL PROFESSION.

EDITED BY J. TAFT AND G. WATT.

The volume commences in January and closes in December.

The Register being issued monthly is always fresh, therefore indispensable to the practitioner who desires to keep posted up with the new inventions and improvements which are daily developed. Neither expense nor effort will be spared to give value and variety to its contents. Articles will be illustrated with well executed engravings whenever they will add to the interest or assist in explanation.

Its extensive circulation and frequency of issue makes it one of the BEST DENTAL ADVERTISING MEDIUMS in the United States.

RATES OF ADVERTISING.

One page, 1 year, $50. Half page, 1 year, $30. Quarter page, or card, 1 year $20.

All advertising bills payable in advance, or at furthest after the issue of the first number containing the advertisement. All transient advertisements to be paid for in advance.

☞ CONTRIBUTIONS SOLICITED.

Address, J. TAFT, or "DENTAL REGISTER," Cincinnati Ohio.

Business Communications to

J. TAFT, Publisher,
No. 117 West Fourth St.

